# Bibliography

**Published:** 1901-08-15

**Authors:** 


### BIBLIOGRAPHY.

A NEW work on "DENTAL ELECTRICITY," by Dr. Levitt E. Custer, has recently been published. The work is a practical statement of the nature of electricity, its sources and management, particularly in its application to the practice of dentistry. While it necessarily follows the plan and uses much of the material found in scientific works on the subject of electricity, it eliminates technical details which would only tend to confuse the dental practitioner, and illustrates the necessary principles by adapting them to familiar appliances.

The introduction is devoted to the subject of *Electricity in Dental Practice*, in which the author demonstrates its wide range of adaptation and the accuracy of its control. It is effective, cleanly, noiseless, uniform in quantity and power, and can be adapted to work without any great amount of machinery.

The first two chapters are given up to a consideration of the nature of electricity and an effort to explain the various scientific terms which have been used in describing its activities. The author has succeeded very well in translating these terms in language or familiar illustrations, which help the novice to get a fairly good conception of their meaning.

About one hundred and twenty pages are devoted to a consideration of the sources of electricity. This is practically one of the most important chapters in the book. It describes and illustrates all varieties of batteries which are used to develop electro-motive force, and also the dynamos and alternators used where larger supplies or varieties of current are wanted. Then comes a discussion of the most favorably known means for controlling the current, the various forms of *Rheostats*. This is a well-considered chapter and offers many practical suggestions of value.

Chapters six, seven and eight are devoted to the application of electricity as a power, and to produce heat and light. The well-known applications are all considered and illustrated and valuable comment made on the various applications made use of by different manufacturers.

A chapter is devoted to *Electrolysis*, and the principles governing this are very well stated, particularly in its adaptation to electro-plating. Some reference is made to its use in therapeutic work, but this branch of dental therapeutics is not sufficiently developed to admit of extended comment. It really seems as though this should be the great use of electricity. The author gives considerable space to the subject of cataphoresis and illustration of appliances. The author concludes that this application of electricity produces the most favorable obtundent results of any method ever used, in spite of the fact that many disastrous results have come from its careless and ignorant use.

The chapter on *The X Ray* is valuable, and seems to promise a practicable appliance for dental office use.

If it can be marketed in portable form and within the financial abilities of the average practitioner it will certainly be a great aid in diagnostic and therapeutic work. The book closes with a description of a private electrical plant for generating and storing electro-motive force. This is a perfectly fitting chapter to close a work of this kind with and we have no doubt the author is capable of installing and utilizing such a plant as he describes, but we are of the opinion that the commercial current will best serve most practitioners.

Like everything Dr. Custer has done, the book is well written, accurate and full of interest, and he has surely placed the dental profession under great obligations for this timely and satisfactory work.

---

THE second edition of Dr. Barrett's "ORAL PATHOLOGY AND PRACTICE" has been issued by the S. S. White Dental Manufacturing Company. The work is much enlarged by the introduction of additional matter and illustrative cuts. Still the author has kept the book well within the bounds of a good student's and practitioner's hand-book. This work should not be classed as a treatise on dental pathology, although it covers the ground fairly well. In our judgment it excels, because of its richness in therapeutic suggestions. We know of no work where so much therapeutic information, briefly stated, can be found. By a systematic classification and paragraphic punctuation the instruction is easily grasped and remembered. It well deserves a wide reading.

